# Supplementary material for: Production of recombinant human IgG1 Fc with beneficial N-glycosylation pattern for anti-inflammatory activity using genome-edited chickens
Source: Commun Biol. 2023 Jun 1;6:589. doi: 10.1038/s42003-023-04937-5 (PMC10235082; doi:10.1038/s42003-023-04937-5)
Supplement: Supplementary file 2 — Supplementary Information [file 42003_2023_4937_MOESM2_ESM.pdf]

Supplementary Information

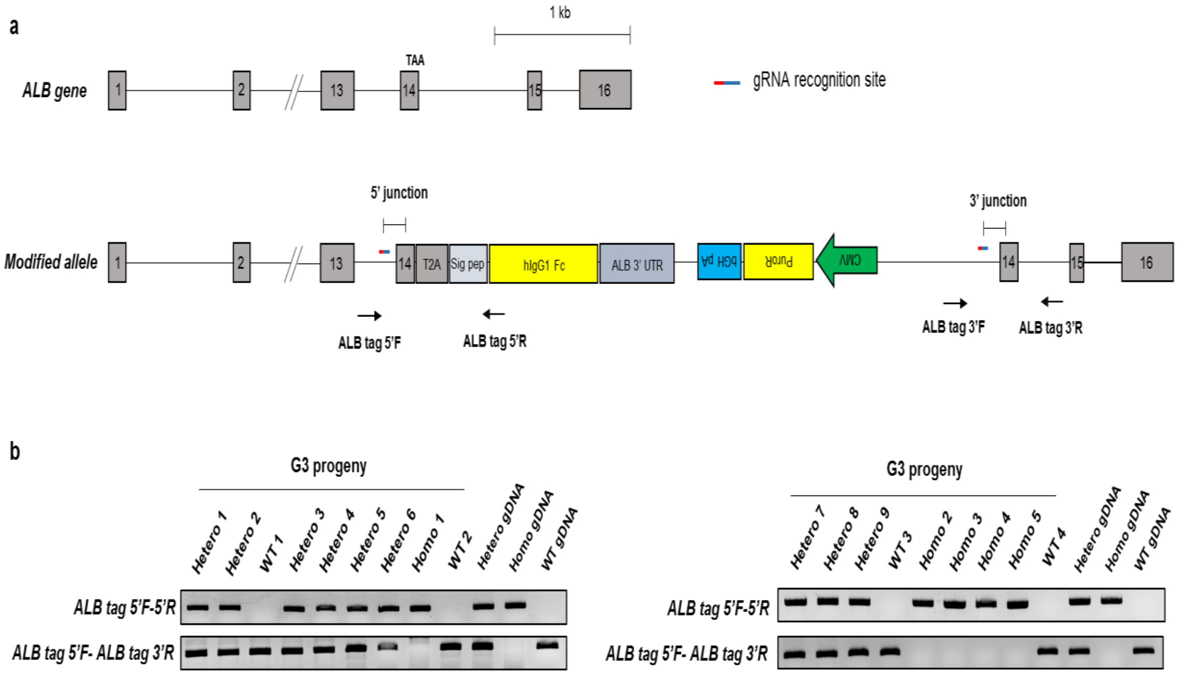

Supplementary Figure 1. Generation of G3 *ALB::hIgG1 Fc* genome-edited progeny. (a) Schematic illustration of the endogenous chicken *ALB* gene and the modified allele of hIgG1 tagged to Fc. Primers specific for the endogenous *ALB* gene and modified allele are denoted by arrows. **(b)** Genotyping of hatched G3 progeny. ALB tag 5’F-5’R primers designed specifically to amplify the modified allele, and ALB tag 5’-ALB tag 3’R primers designed specifically to amplify the endogenous *ALB* gene. Wild-type progeny showed amplicons of the endogenous *ALB* gene only; heterozygous progeny showed amplicons of both endogenous *ALB* gene and the modified allele; homozygous progeny showed amplicons of only the modified allele. Hetero : Heterozygous; Homo : Homozygous; WT : Wild type

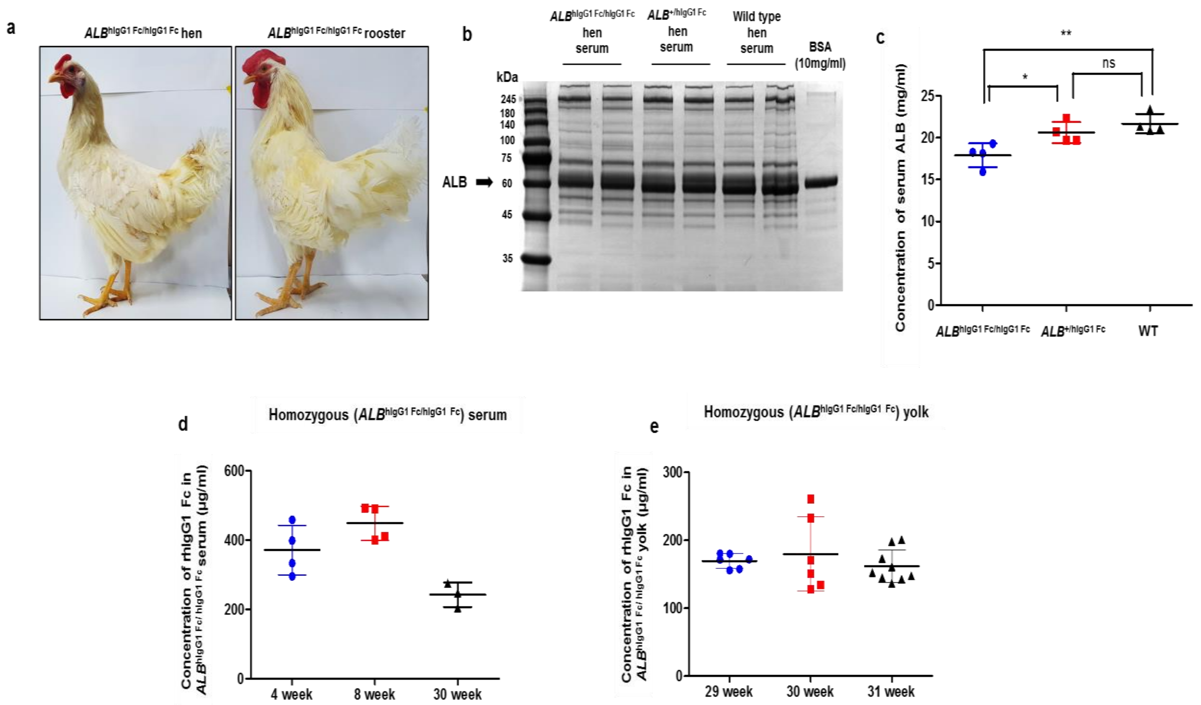

Supplementary Figure 2. Sexual maturation and accumulation of rhIgG1 Fc in serum and egg yolk of homozygous *$ALB^{hIgG1\ Fc/hIgG1\ Fc}$* chickens. (a) Sexually matured 30 weeks after hatching of G3 homozygous hen and rooster. **(b)** SDS-PAGE of homozygous, heterozygous and wild type hen serum. Each serum was 200-fold diluted and stained with Coomassie Brilliant Blue solution. Arrow indicates serum ALB band. **(c)** Estimated concentration of ALB in serum of homozygous, heterozygous and wild type hen using Coomassie Brilliant Blue staining. The concentration was estimated using ImageJ software. 10 mg/ml of bovine serum albumin (BSA) was used as standard for calcualtion. (*n*=4 of independent samples) **(d)** Concentration of rhIgG1 Fc in serum from homozygous *$ALB^{hIgG1\ Fc/hIgG1\ Fc}$* chickens at 4, 8, and 30 weeks after hatching. Each dot represents individual chickens. (*n*=3-4 of independent samples) **(e)** Concentration of rhIgG1 Fc in homozygous *$ALB^{hIgG1\ Fc/hIgG1\ Fc}$* egg yolks at 29, 30, and 31 weeks after hatching. Each dot represent individual eggs from three individual hens. (*n*=6-9 of independent samples) Differences among groups were determined by one-way ANOVA. * $P < 0.05$, ** $P < 0.01$. Error bars represents standard deviation.

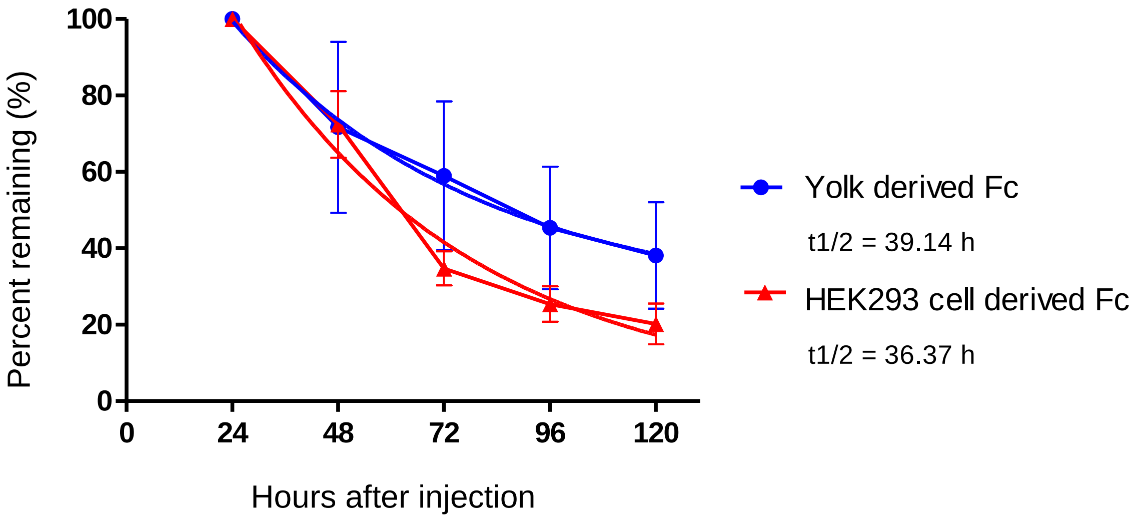

Supplementary Figure 3. In vivo serum half life of rhIgG1 Fc derived from *ALB::hIgG1 Fc* chickens. Clearance of rhIgG1 Fc derived from *ALB::hIgG1 Fc* chicken and recombinant Fc derived from HEK293T cell. Each reagent was i.p. injected into 8 weeks of female C57BL/6 mouse at dose of 100 μg/mouse. After 24 h of injection, serum concentration was measured by ELISA for 5 days. The percent remaining was expressed as percent of 24 h value that the first time-point of measurement (*n*=4 of independent samples). Error bars represents standard deviation.

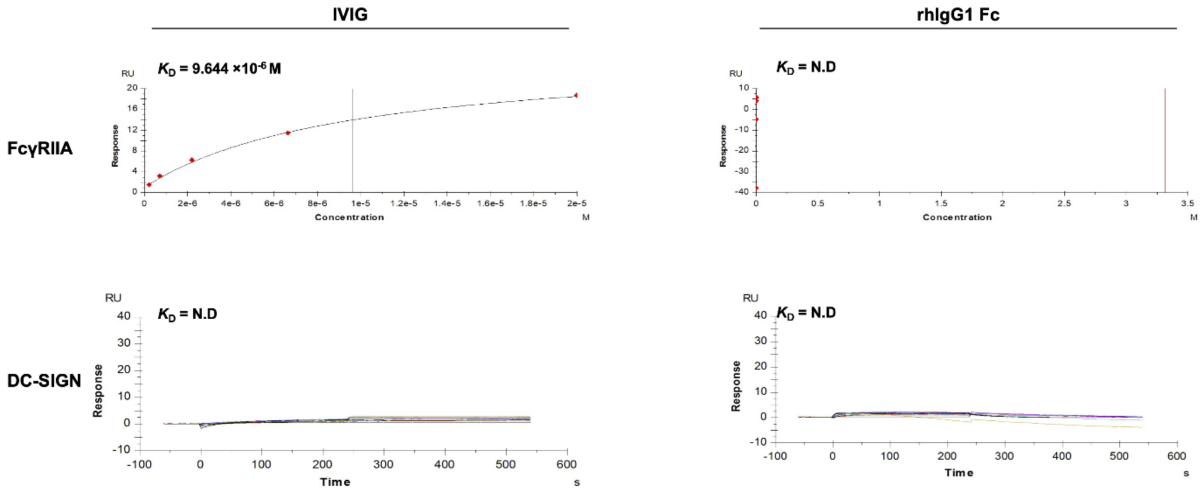

Supplementary Figure 4. Affinity of rhIgG1 Fc purified from *ALB::hIgG1 Fc* chickens for FcγRIIA and DC-SIGN and comparison of that of human IVIG. Sensorgrams showing binding of IVIG and rhIgG1 Fc to hFcγRIIA and DC-SIGN (Biacore analysis). Binding to hFcγRIIA was evaluated using single cycle kinetics and that to DC-SIGN using a steady state equilibrium model.

Supplementary Figure 5. Unprocessed gel image.

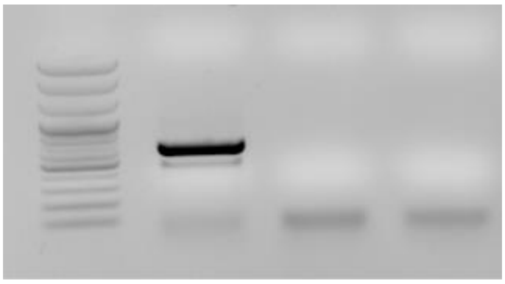

Figure 1b. ALB Tag 5'F-R

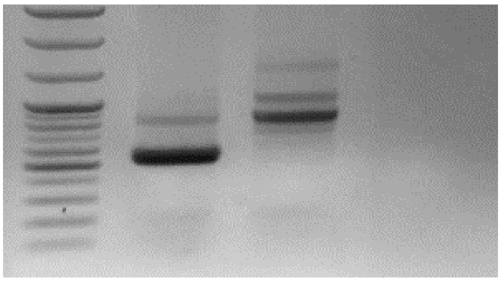

Figure 1b. ALB Tag 3'F-R

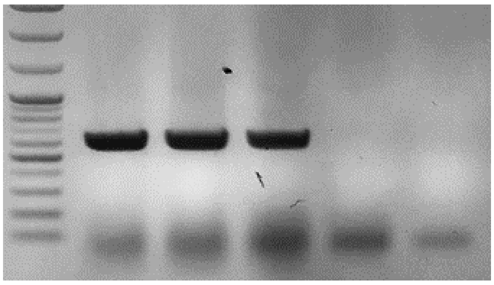

Figure 1e ALB Tag 5'F-R

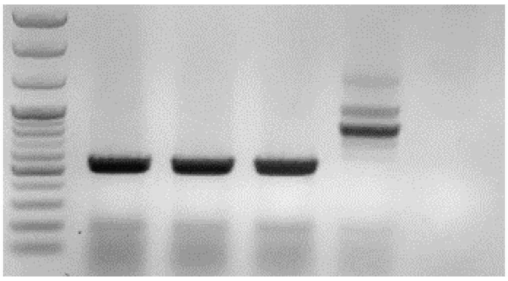

Figure 1e ALB Tag 3'F-R

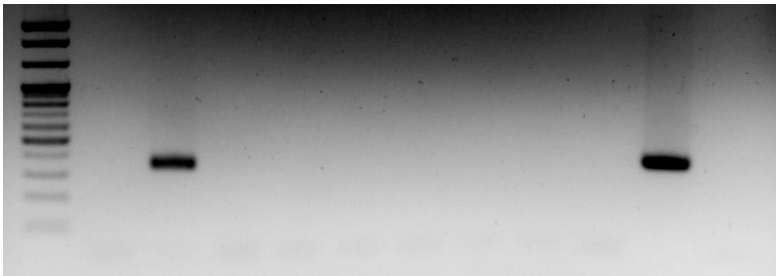

Figure 2a upper panel hIgG1 Fc transcript

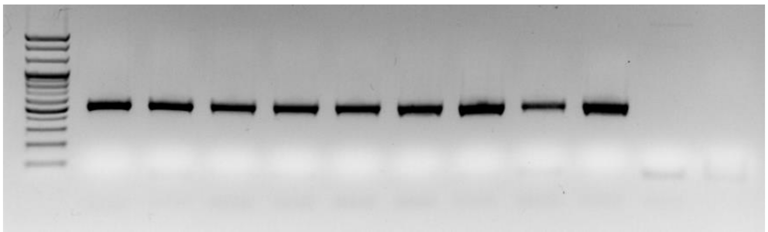

Figure 2a lower panel hIgG1 Fc transcript

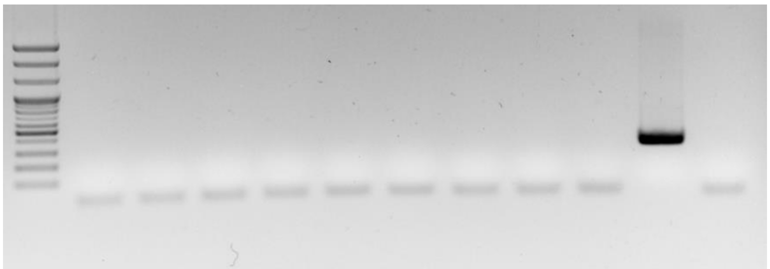

Figure 2a lower panel hIgG1 Fc transcript

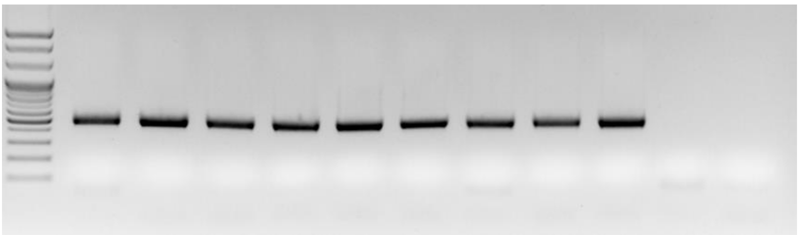

Figure 2a lower panel GAPDH

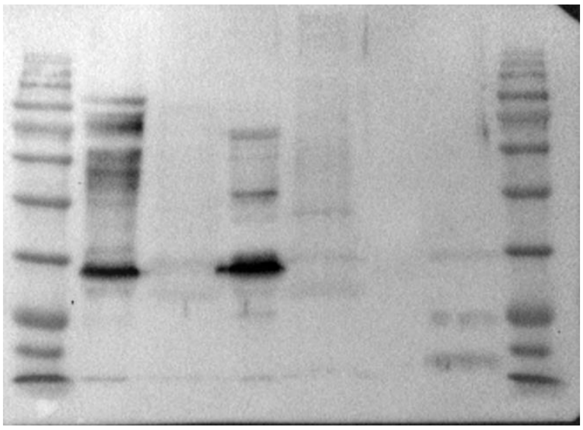

Figure 3e upper panel

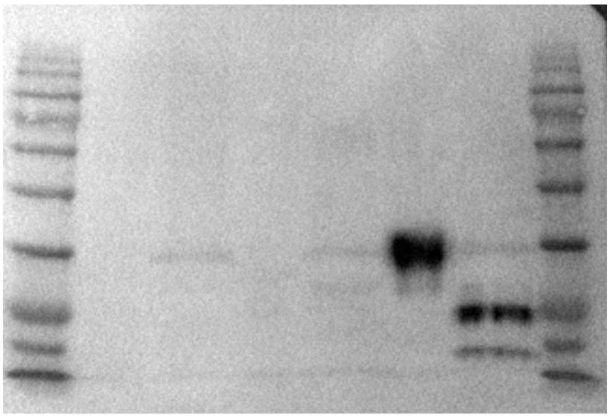

Figure 3e lower panel

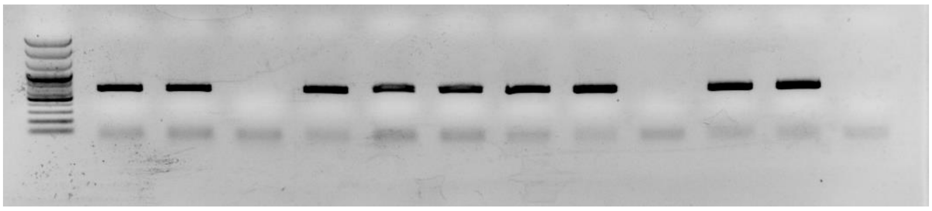

Supplementary Figure 1b upper panel left

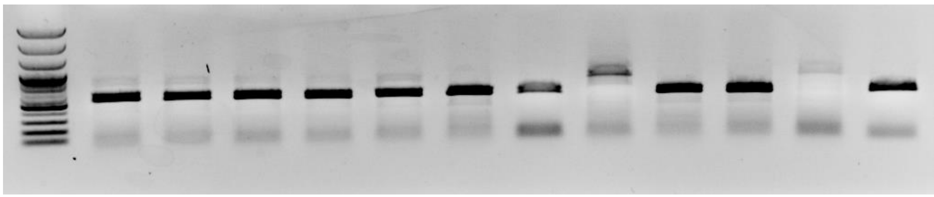

Supplementary Figure 1b lower panel left

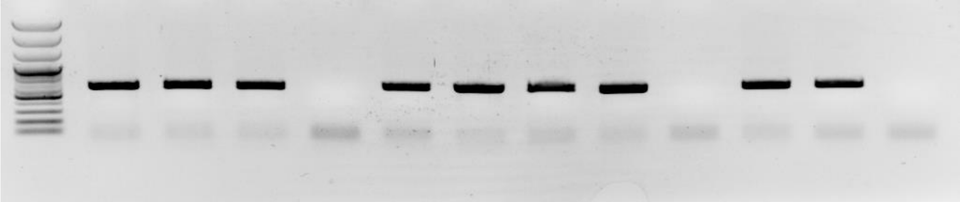

Supplementary Figure 1b upper panel right

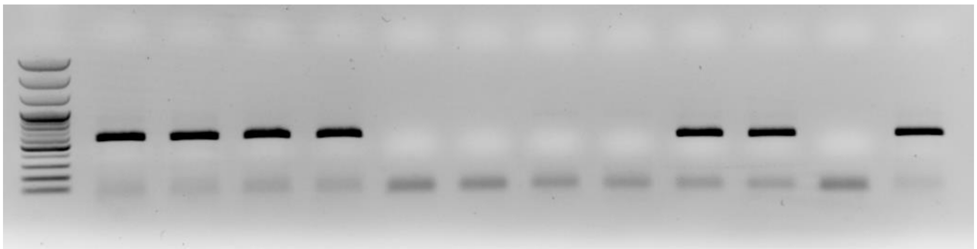

Supplementary Figure 1b lower panel right

Supplementary Table 1. Efficiency of germline transmission and genome edited chick production for *ALB::hIgG1 Fc* donor PGCs

**Wing tag ID**	**Donor cell type**	**No. of hatched chicks**	**No. of endogenous germ-cell–derived chicks* (%)**	**No. of donor germ-cell–derived chicks† (%)**	**No. of *ALB::hIgG1 Fc* chicks (%)‡**
F0357	*ALB::hIgG1 Fc* WL male PGC	75	66 (88.0)	9 (12.0)	6 (66.6)
F0371	*ALB::hIgG1 Fc* WL male PGC	102	100 (98.1)	2 (1.9)	1 (50.0)

* Test-cross analysis was conducted by mating between WL (*I/I*) and germ-line chimeric KO (*i/i*) in which *ALB::hIgG1 Fc* donor PGCs of WL (*I/I*) were transplanted. Therefore, germ-line chimeric KO will produce spermatozoa that derived from endogenous KO germ cells (*i*) and WL germ cells (*I*). The phenotype of progeny derived from endogenous KO germ cell will be hybrid (*I/i*) and derived from donor PGCs will be WL (*I/I*).

† The phenotype of offspring derived from donor PGCs of WL (*I/I*)

‡ The percentage of *ALB::hIgG1 Fc* chicks in donor germ-cell–derived chicks.

Supplementary Table 2. rhIgG1 Fc concentration in serum of G1, G2, G3 heterozygous *ALB::hIgG1 Fc* progenies.

Generation	Individual number	hIgG1 Fc concentration (μg/ml)
**G1**	F0297	133.17
	F0318	114.79
**G2**	F0731	137.09
	F0739	179.41
	F0732	182.63
	F0754	119.58
	F0757	181.09
	F1355	202.21
	F1349	196.85
	F1341	146.61
	F1296	132.24
	F1294	170.88
**G3**	K0755	161.61
	K0757	206.06
	K0762	201.97
	K0765	238.55
	C0764	205.33
	C0775	271.84
	C0767	223.14
	C0769	70.06
	C0773	232.81
	C0776	108.59

Supplementary Table 3. Oligonucleotides used in this study

Name	Sequence
ALB tag 5’ F	5’-GAGCAATCTCTGTCAATGGAAGC-3’
ALB tag 5’ R	5’-ACATGGAGGGCACGTGTGAG-3’
ALB tag 3’ F	5’-AGGGGTTCCGCGCACATTTC-3’
ALB tag 3’ R	5’-GATATTGTAGGCCACCCACTG-3’
hIgG1 Fc RT-F	5’-GCGTCGTGGTGGATGTCTCTC-3’
hIgG1 Fc RT-R	5’-GCTCTCTTGGCTGGCCCTTAG-3’
Chicken GAPDH F	5’-GGTGGCCATCAATGATCCCT-3’
Chicken GAPDH R	5’-TGATGGCATGGACAGTGGTC-3’
Murine FcγRIIB F	5’-CCCTGGGAACTCTTCTACCC-3’
Murine FcγRIIB R	5’-CAGCAGCCAGTCAGAAATCA-3’
Murine IL-33 F	5’-CCTTCTCGCTGATTTCCAAG-3’
Murine IL-33 R	5’-CCGTTACGGATATGGTGGTC-3’
Murine IL-4 F	5’-TCAACCCCCAGCTAGTTGTC-3’
Murine IL-4 R	5’-TGTTCTTCGTTGCTGTGAGG-3’
Murine GAPDH F	5’-CAGAACATCATCCCTGCATCC-3’
Murine GAPDH R	5’-CAGATGCCTGCTTCACCACC-3’
