# Supplementary material for: Production of recombinant human IgG1 Fc with beneficial N-glycosylation pattern for anti-inflammatory activity using genome-edited chickens
Source: Commun Biol. 2023 Jun 1;6:589. doi: 10.1038/s42003-023-04937-5 (PMC10235082; doi:10.1038/s42003-023-04937-5)
Supplement: Supplementary file 3 — Description of Additional Supplementary Files [file 42003_2023_4937_MOESM3_ESM.pdf]

Description of Additional Supplementary Files

File name: Supplementary Data 1

Description: Numerical source data for graphs
