# Supplementary material for: Production of recombinant human IgG1 Fc with beneficial N-glycosylation pattern for anti-inflammatory activity using genome-edited chickens
Source: Commun Biol. 2023 Jun 1;6:589. doi: 10.1038/s42003-023-04937-5 (PMC10235082; doi:10.1038/s42003-023-04937-5)
Supplement: Supplementary file 5 — Reporting Summary [file 42003_2023_4937_MOESM5_ESM.pdf]

nature portfolio

Corresponding author(s): Jae Yong Han

Last updated by author(s): Apr 26, 2023

Reporting Summary

Nature Portfolio wishes to improve the reproducibility of the work that we publish. This form provides structure for consistency and transparency in reporting. For further information on Nature Portfolio policies, see our Editorial Policies and the Editorial Policy Checklist.

Statistics

For all statistical analyses, confirm that the following items are present in the figure legend, table legend, main text, or Methods section.

n/a	Confirmed	
☐	☒	The exact sample size (*n*) for each experimental group/condition, given as a discrete number and unit of measurement
☐	☒	A statement on whether measurements were taken from distinct samples or whether the same sample was measured repeatedly
☐	☒	The statistical test(s) used AND whether they are one- or two-sided<br>*Only common tests should be described solely by name; describe more complex techniques in the Methods section.*
☐	☒	A description of all covariates tested
☒	☐	A description of any assumptions or corrections, such as tests of normality and adjustment for multiple comparisons
☒	☐	A full description of the statistical parameters including central tendency (e.g. means) or other basic estimates (e.g. regression coefficient) AND variation (e.g. standard deviation) or associated estimates of uncertainty (e.g. confidence intervals)
☐	☒	For null hypothesis testing, the test statistic (e.g. *F*, *t*, *r*) with confidence intervals, effect sizes, degrees of freedom and *P* value noted<br>*Give P values as exact values whenever suitable.*
☒	☐	For Bayesian analysis, information on the choice of priors and Markov chain Monte Carlo settings
☒	☐	For hierarchical and complex designs, identification of the appropriate level for tests and full reporting of outcomes
☒	☐	Estimates of effect sizes (e.g. Cohen's *d*, Pearson's *r*), indicating how they were calculated

Our web collection on statistics for biologists contains articles on many of the points above.

Software and code

Policy information about availability of computer code

Data collection	This study does not use any computer software codes for data collection
Data analysis	Statistical analysis of each data were performed using Graphpad Prism (version 9.0) software. Flow cytometry data were analyzed by FlowJo software (version 10.8.0). Chromatography data were analyzed by Waters Empower (version 3.0) software

For manuscripts utilizing custom algorithms or software that are central to the research but not yet described in published literature, software must be made available to editors and reviewers. We strongly encourage code deposition in a community repository (e.g. GitHub). See the Nature Portfolio guidelines for submitting code & software for further information.

Data

Policy information about availability of data

All manuscripts must include a data availability statement. This statement should provide the following information, where applicable:

- Accession codes, unique identifiers, or web links for publicly available datasets
- A description of any restrictions on data availability
- For clinical datasets or third party data, please ensure that the statement adheres to our policy

All data generated during preparatoin of this manuscript are available from the corresponding author on request.

Research involving human participants, their data, or biological material

Policy information about studies with human participants or human data. See also policy information about sex, gender (identity/presentation), and sexual orientation and race, ethnicity and racism.

Reporting on sex and gender	n/a
Reporting on race, ethnicity, or other socially relevant groupings	n/a
Population characteristics	n/a
Recruitment	n/a
Ethics oversight	n/a

Note that full information on the approval of the study protocol must also be provided in the manuscript.

Field-specific reporting

Please select the one below that is the best fit for your research. If you are not sure, read the appropriate sections before making your selection.

☒ Life sciences ☐ Behavioural & social sciences ☐ Ecological, evolutionary & environmental sciences

For a reference copy of the document with all sections, see nature.com/documents/nr-reporting-summary-flat.pdf

Life sciences study design

All studies must disclose on these points even when the disclosure is negative.

Sample size	The size of sample was determined base on the former references in which similar experiments were performed
Data exclusions	There are no data exclusions for analysis
Replication	All experiments are replicated for statistical analysis. The replication number were indicated in the main text, figures and methods section
Randomization	For sample acquisition and analysis from chicken serum and eggs, more than three chickens and eggs at each age were randomly selected. For in vivo analysis, animals were randomly assigned for the different treatment group
Blinding	For experiments of producing genome edited chickens, researchers are not blinded but accumulation of foreign protein in eggs was confirmed so that we did not expect any errors in producing genome edited chickens. For N-glycosylation pattern and SPR analysis, we send samples to independent laboratory out of the university so that we did not expect any bias in data interpretation. For in vivo study, two researchers conduct experiments independently and collected data. In interpreting in vivo data, investigators were not blinded to group allocation

Reporting for specific materials, systems and methods

We require information from authors about some types of materials, experimental systems and methods used in many studies. Here, indicate whether each material, system or method listed is relevant to your study. If you are not sure if a list item applies to your research, read the appropriate section before selecting a response.

Materials & experimental systems

n/a	Involved in the study	
☐	☒	Antibodies
☐	☒	Eukaryotic cell lines
☒	☐	Palaeontology and archaeology
☐	☒	Animals and other organisms
☒	☐	Clinical data
☒	☐	Dual use research of concern
☒	☐	Plants

Methods

n/a	Involved in the study	
☒	☐	ChIP-seq
☐	☒	Flow cytometry
☒	☐	MRI-based neuroimaging

Antibodies

Antibodies used	goat-anti human IgG primary antibody (10319, Alpha diagnostic), horseradish peroxidase (HRP)-conjugated donkey anti-goat IgG

Antibodies used	secondary antibody (sc-2020, Santa Cruz Biotechnology), Sambucus Nigra Lectin (SNA, EBL), Biotinylated (B-1305-2, Vector Laboratories), Maackia Amurensis Lectin II (MAL II), Biotinylated (B-1265-1, Vector Laboratories), horseradish peroxidase-conjugated avidin (VECTASTAIN ABC kit, Vector Laboratories)
Validation	All primary and secondary antibodies and lectins used in this study are commercially available and validated by manufacturers

Eukaryotic cell lines

Policy information about cell lines and Sex and Gender in Research

Cell line source(s)	Chicken primordial germ cells, WIL2-S, Raji , Jurkat T cell
Authentication	Chicken primordial germ cell lines are established in our laboratory. WIL2-S, Raji and Jurkat T cells are provided by ADCC and ADCP reporter bioassay kits (PROMEGA)
Mycoplasma contamination	We confirmed that all cells used in this manuscript are free of mycoplasma contamination
Commonly misidentified lines (See ICLAC register)	There are no commonly misidentified cell lines used in this study

Animals and other research organisms

Policy information about studies involving animals; ARRIVE guidelines recommended for reporting animal research, and Sex and Gender in Research

Laboratory animals	C57BL/6 mouse, Female, 8 weeks of age<br>White Leghorn chickens, Both sexes, 12~40 weeks after hatch
Wild animals	Wild animals were not involved in this study
Reporting on sex	Female mice were used for in vivo experiments. Analysis of recombinant Fc was performed from both sexes of chicken
Field-collected samples	Field-collected samples were not involved in this study
Ethics oversight	The management and experimental uses of animals were approved by the Institutional Animal Care and Use Committee (IACUC), Seoul National University

Note that full information on the approval of the study protocol must also be provided in the manuscript.

Flow Cytometry

Plots

Confirm that:

☒ The axis labels state the marker and fluorochrome used (e.g. CD4-FITC).

☒ The axis scales are clearly visible. Include numbers along axes only for bottom left plot of group (a 'group' is an analysis of identical markers).

☒ All plots are contour plots with outliers or pseudocolor plots.

☒ A numerical value for number of cells or percentage (with statistics) is provided.

Methodology

Sample preparation	Spleen of mouse in each experimental groups were extracted after 6 hour of sample treatment. Spleens were dissociated into single cells by using Trypsin-EDTA. Dissociated spleen cells were immunostained using a mouse Treg detection kit (130-120-674, Miltenyi Biotec). After that, the spleen cells were stained with APC labeled anti mouse CD25 antibody and Vio-Blue-labeled CD4 antibody for 30 min under refrigeration. Cells were then washed with PBS, permeabilized, and fixed using a fixation/permeabilization solution for 30 min in dark under refrigeration. After washing twice, the cells were treated with PE-labeled anti-mouse Foxp3 antibody for 30 min in dark under refrigeration
Instrument	Flow Cytometer (BD bioscience)
Software	FlowJo (version 10.8.0) Software (Tree Star)
Cell population abundance	More than 10,000 cells were counted per sample. No sorting was conducted

Gating strategy

Analysis was performed with FSC/SSC gating to remove debris and dead cells. To remove doublets and other aggregated particels, single cell gating was performed by FSC-A x FSC-H parameter. Gating was determined using unstained control and single-stained controls (CD4 and FOXP3) to set up flow cytometer compensatio and quadrants.

☒ Tick this box to confirm that a figure exemplifying the gating strategy is provided in the Supplementary Information.
